# Body mass index and fat influences the role of *Bifidobacterium* genus in lupus patients concerning fibrinogen levels

**DOI:** 10.3389/fmicb.2024.1471177

**Published:** 2024-11-25

**Authors:** Lourdes Chero-Sandoval, Andrea Higuera-Gómez, Amanda Cuevas-Sierra, Begoña de Cuevillas, Raquel Castejón, María Martínez-Urbistondo, Susana Mellor-Pita, Víctor Moreno-Torres, Daniel de Luis, J. Alfredo Martínez

**Affiliations:** ^1^Precision Nutrition and Cardiometabolic Health, IMDEA-Food Institute (Madrid Institute for Advanced Studies), Campus of International Excellence (CEI) UAM+CSIC, Madrid, Spain; ^2^Department of Endocrinology and Nutrition, University Clinical Hospital, University of Valladolid, Valladolid, Spain; ^3^Health Sciences School and Medical Centre, International University of the Rioja (UNIR), Madrid, Spain; ^4^Internal Medicine Service, Puerta de Hierro Majadahonda University Hospital, Madrid, Spain; ^5^Centre of Endocrinology and Nutrition, University of Valladolid, Valladolid, Spain; ^6^CIBERobn Physiopathology of Obesity and Nutrition, Institute of Health Carlos III (ISCIII), Madrid, Spain

**Keywords:** *Bifidobacterium*, body mass index, fibrinogen, low-grade metabolic inflammation, systemic lupus erythematosus

## Abstract

**Introduction:**

Metabolic disorders and autoimmune diseases elicit distinct yet interconnected manifestations of inflammation, which may be boosted by an excess of body adiposity. The purpose of this investigation was to analyze anthropometric, biochemical, and inflammatory/coagulation variables concerning patients diagnosed with systemic lupus erythematosus (SLE) exploiting low-grade metabolic inflammation (MI), as reference.

**Methods:**

A population stratification by body mass index (BMI), allowed to assess the impact of adiposity on the putative role of gut microbiota composition on coagulation markers. A total of 127 participants with MI and SLE were categorized into two main groups based on their BMI, following WHO criteria: a low BMI group (<30 kg/m^2^) and a high BMI group (≥30 kg/m^2^). Each group included recorded data on demographics, comorbidities, and key clinical markers. Anthropometric and body composition variables, clinical features, and inflammatory/coagulation markers were measured while fecal 16S rRNA sequencing was examined at the genus *Bifidobacterium*. Regression models were fitted to evaluate the relationship between gut microbiota, inflammatory/coagulation markers, and body weight in these types of diseases.

**Results:**

The study revealed worse clinical outcomes in anthropometric, body composition, and clinical markers in low-grade MI conditions as compared to SLE. However, inflammatory and coagulation markers such as C-reactive protein (CRP) and fibrinogen were significantly more elevated in patients with SLE, which was exacerbated by high BMI/ body fat as compared to the other screened groups. An interaction analysis revealed that fibrinogen levels showed different trends when *Bifidobacterium* was increased depending on BMI/adiposity, which evidenced an effect modification by this microorganism in patients with SLE.

**Discussion:**

These findings underline that gut microbiota composition, particularly the presence of *Bifidobacterium*, may play a crucial role in modulating inflammation and coagulation processes in patients with SLE and high fat. These insights highlight the potential of targeting gut microbiota as a therapeutic strategy to mitigate inflammation and improve clinical outcomes in SLE patients.

## 1 Introduction

Chronic metabolic disorders and autoimmune diseases are marked by complex interactions between inflammatory processes, immune system alterations, coagulation disorders and metabolic problems (Uddin et al., 2022). Thus, autoimmune diseases such as systemic lupus erythematosus (SLE) and rheumatoid arthritis are characterized by abnormal immune responses directed against self-antigens, resulting in tissue damage and cell dysfunction (Szekanecz et al., 2021). Furthermore, in addition, inflammatory disorders can manifest systemically, affecting multiple organ systems, while metabolic disorders, such as fat accumulation, insulin resistance, and dyslipidemia, reflect adverse biochemical, immunological, and pathophysiological outcomes (Khanna et al., 2022).

Recent studies highlight the important role of adiposity in exacerbating inflammation and altering immune responses in patients with SLE and metabolic disorders (Geng et al., 2022). Excess body weight contributes to a state of chronic low-grade inflammation, characterized by elevated levels of proinflammatory cytokines such as tumor necrosis factor-*α* (TNF-α), interleukin-6 (IL-6) and interleukin-1β (IL-1β) (Geng et al., 2022; Karczewski et al., 2021). In addition, visceral fat accumulation has been linked to increased intestinal permeability and dysbiosis, which can perpetuate systemic inflammation and metabolic dysregulations (Wang et al., 2022; Xu et al., 2022). This chronic inflammatory state is also often associated with alterations in liver function and other pathophysiological processes, further dysregulating coagulation, systemic inflammation, and immune responses (Pietzner et al., 2017). In addition, excess body weight has been closely related to alterations in gut microbiota composition and function (Turnbaugh et al., 2006).

In this context, gut microbiota assessment has emerged as a potential tool in the prognosis and diagnostic examination of various diseases, highlighting a specific role in health status (Duan et al., 2024). Dysbiosis, characterized by a putative imbalance in gut microbial communities, has been implicated in the pathogenesis of SLE, affecting both disease severity and overall inflammatory status (Zeng et al., 2017; Zhao et al., 2023). Such an imbalance is associated with increased intestinal permeability, microbial translocation, and release of proinflammatory molecules such as lipopolysaccharides (LPS) into the systemic circulation, thereby perpetuating inflammation and exacerbating metabolic dysfunctions (Geng et al., 2022; Wang et al., 2020; Zeng et al., 2017). Specific bacterial genera, such as *Bifidobacterium*, have been associated with positive health outcomes and may play a protective role against inflammation and autoimmune responses (Duan et al., 2024). Conversely, reduced diversity and abundance of beneficial gut bacteria may exacerbate the inflammatory milieu in SLE patients (Zeng et al., 2017).

Hypercoagulation is common in SLE, and elevated fibrinogen levels reflect both inflammation and an increased risk of thrombosis in these patients (Sulimai et al., 2022; Wolberg, 2023). Fibrinogen is an acute phase reactant that increases in response to injury and disease associated with inflammatory conditions (Cicarini et al., 2020; Sulimai et al., 2022). Also, fibrinogen monitoring in SLE patients has been shown critical to assess cardiovascular risk, guide SLE treatment and manage chronic inflammation associated with the disease (Cicarini et al., 2020; Zadeh et al., 2020). Other markers, such as D-dimer, are also altered in these SLE patients being associated with a hypercoagulation state and increased risk of venous thromboembolism, underlining the importance of comprehensive coagulation monitoring in this autoimmunity population (Zinellu and Mangoni, 2024).

Holistic interventions that focus on dietary modifications, microbiota modulation, and body weight control show promise in precision nutrition by potentially enhancing clinical outcomes through the restoration of gut microbiota balance and reduction of inflammation (Dasriya et al., 2024). By exploring the interactions among inflammation, coagulation, and gut microbiota composition in affected individuals, precision medicine strategies can be tailored to meet the unique needs of each patient, ultimately leading to more effective management and improved clinical outcomes with integrated information (Ball and Athanasiadou, 2021).

The primary objective of this research was to investigate clinical characteristics, adiposity metrics, biochemical markers, and inflammation/coagulation biomarkers in patients with SLE compared to those with low-grade metabolic inflammation (MI), analyzing these features in relation to body mass index (BMI). Additionally, we aimed to explore the potential role of the gut microbiota, particularly to identify similarities and differences in coagulation pathways impacted by SLE and MI. Specifically, we hypothesized that there are significant interactive effects (effect modification) between *Bifidobacterium* levels and BMI on coagulation markers, such as fibrinogen, in SLE patients. By elucidating these relationships, this study aims to contribute to a deeper understanding of gut microbiota’s impact on inflammation and coagulation in SLE, advancing the development of targeted interventions for optimized patient outcomes.

## 2 Materials and methods

### 2.1 Study design

This study is part of the “METAINFLAMMATION” project (ref. Y2020/BIO-6600), a prospective and controlled investigation. Participant recruitment occurred between January 2022 and June 2023 at the Internal Medicine Service of Puerta de Hierro Majadahonda University Hospital in Madrid, Spain. The enrollment process involved obtaining participant agreement for inclusion in the study and ensuring the completion of informed consent forms. The study was conducted in compliance with the Declaration of Helsinki principles and received approval from the Research Ethics Committee of Puerta de Hierro Majadahonda University Hospital (file number PI 164–21). All data collection was performed following ethical guidelines and strict hospital protocols.

### 2.2 Participants, inclusion, and exclusion criteria

This research involved 127 participants, both men (28%) and women (72%) of Caucasian/Hispanic ancestry, which is considered adequate to meet the stated analytical objectives, based on the existing literature and prior project data, a sample size of 127 participants is suited to meet the analytical objectives of this study (Martinez-Urbistondo et al., 2020). In the development of the METAINFLAMMATION project, weight was initially used as the primary variable, setting a target sample size of 80 participants. Additionally, when an *a posteriori* calculation was conducted to determine the necessary sample size with fibrinogen (mg/dL) as the primary variable and assuming a mean difference of 50, a standard deviation of 100, a significance level (*α*) of 0.05, and a power (*β*) of 80%, it was estimated that a total sample size of 100 participants is required, further supporting the sufficiency of the current sample for the study’s objectives. The participants were assigned into two groups according to the medical diagnoses received: low-grade MI and SLE, both diseases are considered complex conditions that are characterized by an inflammatory state (Xiao et al., 2021). The low-grade MI refers to a chronic subclinical inflammatory state that occurs in the absence of acute infection which is often found in excessive weight conditions (Khanna et al., 2022; Van de Vyver, 2023). This phenomenon results largely from metabolic dysregulation, common in conditions such as obesity and related metabolic disorders, leading to sustained immune activation (Polak-Szczybyło, 2023; Terkawi et al., 2022). Concerning our analysis, we grouped together normoweight and overweight individuals to capture how metabolic inflammation may be consistently present across different body weight profiles, while excluding clinical obesity. This classification aligns with medical literature indicating that metabolic inflammation can occur even in overweight individuals who are not classified as obese, potentially serving as an early indicator for metabolic disease development (Hotamisligil, 2006; Lumeng and Saltiel, 2011). In terms of low BMI, we defined this as a BMI under 30 kg/m^2^, consistent with WHO criteria, which categorize individuals below this threshold as non-obese but overweight if BMI > 25 kg/m^2^. The diagnostic criteria established by the World Health Organization (WHO) and the National Education Program on Cholesterol (NECP) were used to identify patients with obesity and metabolic syndrome (World Health Organization, 2024), while for the SLE group, the classification criteria established by the European League Against Rheumatism/American College of Rheumatology were applied (EULAR/ACR) (Aringer et al., 2019). Body weight stratification was performed using the definition of BMI according to the World Health Organization (WHO) (World Health Organization, 2024) criteria, which establishes obesity with a BMI of ≥30 kg/m^2^. The groups were subdivided into two categories: low BMI (≤ 30 kg/m^2^) and high BMI (≥30 kg/m^2^).

The participants met the following inclusion criteria: age > 18 years, a body mass index >18 kg/m^2^ and < 50 kg/m^2^ and diagnosis of low-grade MI and SLE confirmed by the medical staff of the Internal Medicine service of the Puerta de Hierro Majadahonda University Hospital (Madrid, Spain). Patients with obesity and metabolic syndrome presented a series of alterations such as excessive adiposity, glucose intolerance, central obesity, dyslipidemia, and hypertension according to adult treatment panel III criteria (ATP III) (Khanna et al., 2022). Meanwhile, patients diagnosed with SLE in a stable state and under protocolized supervised medical treatment were selected, which allows us to minimize biases related to disease activity and to provide a comprehensive understanding of the cohort features and the homogeneity of the patient population. All patients followed a uniform treatment protocol, and no relevant differences were observed between patients. In addition, the impact of corticosteroids in these patients is minimal due to controlled doses and disease stability, which significantly reduces their influence on the results. It should be noted that corticosteroids were administered only in acute situations and not as a persistent treatment. Several clinical variables were assessed in these patients, including serological activity (SA), presence of active disease (AD), achievement of complete remission (CR) and maintenance of a low disease activity state (LDAS) to harmonize the clinical diagnosis and therapeutical homogeneity of the SLE population (Gladman et al., 1997; Moreno-Torres et al., 2022). Exclusion criteria included the presence of severe psychiatric disorders, the current use of body weight-modifying agents, difficulty for scheduling appointments, pregnancy, lactation, and patients with changes in pharmacological prescription one year before. Participants who regularly consumed probiotics or recent antibiotics at least 3 weeks before the collection of the fecal sample were also excluded.

### 2.3 Anthropometrics and clinical data

Anthropometric measurements were assessed by skilled dietitians using validated techniques (Martínez Urbistondo et al., 2021). Body weight was determined using a bioimpedance scale (TANITA SC-330; Tanita Corporation Pais), which also provided (absolute or relative) estimates of body composition (skeletal muscle mass, body fat and visceral fat). Waist circumference was measured with a standard tape measure following established protocols and performed by trained dietitians, while BMI was calculated as the ratio of body weight to the square of height (kg/m^2^) as described elsewhere (Martínez Urbistondo et al., 2021).

### 2.4 Biochemical data

Blood samples were assessed under fasting conditions through venipuncture. The samples underwent analysis for platelets and red cell distribution width (RDW) utilizing an SYSMEX XN-20 automated hematology analyzer (Roche, Basel, Switzerland) following validated procedures. Routine biochemical markers, including glucose, total cholesterol, glycated hemoglobin, folic acid, bilirubin, vitamin D, ferritin, high density lipoprotein (HDL), low density lipoprotein (LDL), triglycerides, and transferrin, were measured following standardized hospital protocols using a quality-controlled autoanalyzer (Atellica™ Solution Pais) as per established criteria (Martinez-Urbistondo et al., 2020). C-reactive protein (CRP), fibrinogen, insulin, interleukin-6 (IL-6), and D-dimer also followed analytically standardized procedures, primarily employing specific ELISA kits (Sigma-Aldrich ELISA Kit Pais) as outlined by the suppliers.

### 2.5 Metagenomic analysis

Fecal samples were collected using OMNIgene® •GUT kits (DNA Genotek, Ottawa, ON, Canada), according to the supplier instructions (Cuevas-Sierra et al., 2022). Bacterial DNA was isolated with the QIAamp® DNA kit (Qiagen, Hilden, Germany) following the manufacturer’s protocol and the V3-V4 hypervariable regions of the 16 S rRNA gene were amplified by paired-end DNA sequencing in the MiSeq System (Illumina, San Diego, CA, USA) at Novogene Sequencing- Europe Service (Cambridge, United Kingdom). Also, the primers used for the PCR reactions were (16S Amplicon PCR Forward Primer = 5 0 TCGTCGGCAGCGTCAGATGTGTATAAGAGACAGCCTACGGGNGGCWGCAG, 16S Amplicon PCR Reverse Primer = 5 0 GTCTCGTGGGCTCGGAGATGTGTATAAGAGACAGGACTACHVGGGTATCT AATCC). PCR reactions were carried out with 15 μL of Phusion® High – Fidelity PCR Master Mix (New England Biolabs); 0.2 μM of forward and reverse primers, and about 10 ng template DNA. Thermal cycling consisted of initial denaturation at 98°C for 1 min, followed by 30 cycles of denaturation at 98°C for 10 s, annealing at 50°C for 30 s, and elongation at 72°C for 30 s and 72°C for 5 min. The PCR products were purified using magnetic beads and the samples were mixed in equidensity ratios based on the concentration of PCR products. After thorough mixing, the PCR products were detected, and target bands were recovered. For library preparation, sequencing libraries were generated, and indexes were added. The library was checked with Qubit and real-time PCR for quantification and bioanalyzer for size distribution detection. Quantified libraries were pooled and sequenced on Illumina platforms, according to effective library concentration and data amount required. For bioinformatic analysis, paired- end reads were assigned to samples based on their unique barcode and truncated by cutting off the barcode and primer sequence. Paired-end reads were merged using FLASH V1.2.7 (USA^1^) (Magoč and Salzberg, 2011), while quality filtering on the raw tags were performed using the FASTP V0.23.1 (China, FASTP Software^2^) to obtain high-quality clean tags (Bokulich et al., 2013). The tags were compared with the reference Silva database (16S/18S^3^; UNITE Database^4^) to detect chimera sequences, and then the chimera sequences were removed (Frank et al., 2011). For the effective tags obtained previously, denoise was performed with DADA2 or deblur module in the QIIME2 software (Version QIIME2-202202, USA^5^) to obtain initial ASVs (Amplicon Sequence Variants). Species annotation was performed using QIIME2 software (SILVA Database) and to study phylogenetic relationship of each ASV and the differences of the dominant species among different samples (groups), multiple sequence alignment was performed using QIIME2 software and displayed with R software (Version 2, USA^6^) (Bolyen et al., 2019).

### 2.6 Statistical analyses

Variables were expressed as means (x̄) and standard deviations (SD) for quantitative variables and number of cases (*n*) and proportions (%) for qualitative variables. Normality of the data was assessed by the Shapiro–Wilk test. Student’s *t* and Mann–Whitney tests were implemented depending on normality to compare the means of the continuous variables at the beginning of the study and the categorical variables were statistically screened using the chi-square (χ^2^) test. BMI was used as a proxy of adiposity in some analyses, since this estimation showed a high correlation with body fat (R^2^ = 0.7433 and *p* value<0.001) and has a higher value for translational purposes. While body fat offers a more direct measure of adiposity and related effects, BMI was chosen for its ease of interpretation in clinical and research settings. The differences and interactions between the two types of diseases and the BMI stratified by low BMI (≤ 30 kg/m^2^) and high BMI (≥30 kg/m^2^) were studied with a 2 × 2 factorial ANOVA design (2 diseases x 2 levels of BMI) for anthropometric variables, body composition, biochemical markers, inflammatory and coagulation features concerning METAINFLAMMATION study participants by RStudio 4.3.0 [University of Auckland, New Zealand (see Footnote 6)]. The analysis of microbiota was evaluated comparing the two types of inflammatory diseases, adjusted by BMI levels, age and sex to avoid potential confounders. Alpha diversity was calculated by determining the Shannon index using MicrobiomeAnalyst 2.0 (University of McGill, Canada^7^) (Dhariwal et al., 2017; Konopiński, 2020) and compared by *t*-test and illustrated with boxplots. Beta diversity was calculated using Bray Curtis index and PERMANOVA test and then visualized by means of principal coordinate analysis (PCoA). In addition, linear discriminant analysis (LDA) effect size (LEfSe 1.0) (Harvard University; USA^8^) was used to compare groups and to report the results using taxonomic bar charts. Zero-inflated Gaussian (metagenomeSeq) analysis was for finding families that differed significantly in abundance between normal body weight and obese subjects. Differential abundance analysis was performed using EdgeR method, and FDR correction (Trimmed- mean of M value for normalization). Random forest was used to rank the importance of predictive variables related to type of disease using R 3.5.3 [University of Auckland, New Zealand (see Footnote 6)] and Receiver Operating Characteristic curve (ROC) was performed to validate the Random Forest results. To enhance data reliability, a low count filter was applied to remove features with limited prevalence or low abundance across samples. Specifically, features with fewer than four observations were excluded, and only those present in at least 20% of the samples were retained. This filtering process reduced the dataset from the initial 458 sequenced genera to 311, focusing the analysis on consistently observed taxa and minimizing noise from rare or low-abundance features. A random forest model was then applied using 500 trees, where feature importance was assessed using the “mean decrease in accuracy” metric. This metric evaluates each feature’s impact on prediction accuracy by measuring the error increase when a feature is removed or permuted. For clarity, only the top 10 most impactful features were presented graphically to ensure readability. Potential interactions between bacteria, type of disease and inflammatory status were investigated with multiple regression models adjusted for age, sex, BMI, disease, *Bifidobacterium*, *Bifidobacterium adolescentis, Bifidobacterium longum,* and the interaction between bacteria and BMI levels using RStudio 4.3.0 (USA) (see Footnote 6) and for graphs of interaction between *Bifidobacterium* abundance, fibrinogen levels, and both BMI and body fat levels in SLE patients, Stata 12 (StataCorp LLC, College Station, TX, USA^9^) was used. The stratification of body fat was carried out taking as reference the average, low fat <36 and high fat ≥36. Normalization of the microbiota data was performed according to the centered log ratio (CLR) method, to ensure reliable, meaningful results in our analysis of microbiota and its associations with health outcomes, as it has been established as best practice for compositional data (Gloor et al., 2017). This approach not only aligns with rigorous statistical standards but also reinforces the validity of our findings (Gloor et al., 2017; Palarea-Albaladejo and Martín-Fernández, 2015). A value of *p* < 0.05 was considered statistically significant.

## 3 Results

### 3.1 Comparison of anthropometric, body composition, biochemical, and clinical markers in patients with low-grade MI and SLE according to BMI levels

Table 1 shows the distinct anthropometric and body composition profiles between patients with low-grade MI and SLE stratified by BMI levels. Participants with high BMI showed significantly higher values for all variables in both types of disease. In addition, in the SLE group, skeletal muscle did not present significant differences when compared according to BMI. The comparison of variables according to the group of disease without considering BMI status (Group of disease column, Table 1) showed significant differences between groups of disease for these variables in this population, excepting in body fat, while the comparison of variables according to the BMI without considering the type of disease (BMI levels column, Table 1) showed significant differences between groups of BMI for these variables in this population, excepting in gender distribution and the interaction effect between groups of disease and BMI levels (Interaction column, Table 1) was significant for body fat and waist circumference, showing a modification of the effect of BMI on these anthropometric variables according to the type of inflammatory disease.

**Table 1:** Comparison of anthropometric measurements and body composition between types of inflammatory conditions (low-grade MI and SLE) and according to groups of BMI status.

Variables	Hospital reference values	Low-grade metabolic inflammation (MI)			Systemic lupus erythematosus (SLE)			*p* value		
		Low BMI (*n* = 24)	High BMI (*n* = 46)	*p* value	Low BMI (*n* = 39)	High BMI (*n* = 18)	*p* value	Group of disease	BMI levels	Interaction
Age (years)	NA	59.9 (9.1)	59.2 (11.2)	0.76	50.1 (11.5)	56.7 (10.8)	0.04	**<0.001**	**0.02**	0.08
Gender = Woman (%)	NA	14 (58.3)	25 (54.3)	0.95	35 (89.7)	18 (100.0)	0.39	**<0.001**	0.18	0.37
Body weight (Kg)	NA	74.8 (8.8)	96.5 (15.9)	**<0.001**	65.8 (9.6)	87.8 (16.9)	**<0.001**	**<0.001**	**<0.001**	0.95
Body mass index (Kg/m^2^)	18.5–24.9	27.7 (1.4)	34.2 (3.2)	**<0.001**	24.9 (3.1)	34.7 (3.8)	**<0.001**	**<0.001**	**<0.001**	**<0.01**
Waist circumference (cm)	Male <94 Female <80	100.9 (6.0)	115.3 (9.6)	**<0.001**	90.0 (9.6)	111.5 (9.2)	**<0.001**	**<0.001**	**<0.001**	**0.02**
Skeletal muscle mass (Kg)	NA	47.9 (7.4)	55.1 (11.8)	**0.02**	42.9 (5.8)	45.6 (7.4)	0.22	**<0.001**	**<0.001**	0.18
Body fat (%)	NA	32.8 (4.3)	40.0 (6.6)	**<0.001**	30.9 (6.3)	45.0 (4.1)	**<0.001**	0.12	**<0.001**	**<0.01**
Visceral fat (AU)	NA	10.6 (3.3)	15.9 (4.5)	**<0.001**	6.7 (3.7)	12.4 (2.1)	**<0.001**	**<0.001**	**<0.001**	0.82

Data presented as mean (x̄), standard deviation (SD), and *p* values. The significance threshold was set at *p* < 0.05 *. *p* value refers to the comparison of variables’ mean between patients with low-grade MI and SLE using *t*-test or Mann–Whitney test, according to the distribution of the data. Low BMI refers to BMI ≤ 30 kg/m^2^ and high BMI refers to BMI ≥ 30 kg/m^2^. “Group of disease” column is the comparison of variables’ mean between the two inflammatory diseases (without considering BMI status). “BMI levels” column is the comparison of variables’ mean between the low and high BMI. Interaction column means the *p* value of comparison between the two disease groups and the BMI levels (ANOVA 2×2). AU, Arbitrary units. *p* value lower than 0.05 are in bold type.

Table 2 shows the comparison of biochemical profiles between patients with low-grade MI and SLE, according to the type of disease and BMI levels. Patients with low-grade MI and high BMI displayed significantly higher levels of insulin levels, bilirubin, and vitamin D. On the contrary, SLE patients with increased BMI exhibited significantly higher levels of triglycerides, glycated hemoglobin, insulin, and transferrin. Furthermore, subgroup analysis based on the group of disease without considering BMI (Group of disease column, Table 2) showed significant differences in glucose, HDL-cholesterol, glycated hemoglobin, and bilirubin, being higher in low-grade MI group, excepting in HDL-cholesterol, which was higher in SLE group.

**Table 2:** Comparison of biochemical and clinical markers between low-grade MI and SLE participants and according to groups of BMI of the METAINFLAMMATION cohort.

Variables	Hospital reference values	Low-grade metabolic inflammation (MI)			Systemic lupus erythematosus (SLE)			*p* value		
		Low BMI (*n* = 24)	High BMI (*n* = 46)	*p* value	Low BMI (*n* = 39)	High BMI (*n* = 18)	*p* value	Group of disease	BMI levels	Interaction
Glucose (mg/dL)	60–100	103.8 (18.6)	99.6 (13.5)	0.55	88.8 (8.2)	93.2 (17.2)	0.53	**<0.001**	0.25	0.11
Insulin (μUI/mL)	0–29.1	8.0 (2.9)	13.6 (9.0)	**0.01**	8.3 (5.7)	14.4 (9.8)	**0.002**	0.28	**<0.001**	0.87
Glycated Hemoglobin (%)	4.5–6.4	5.7 (0.5)	5.6 (0.6)	0.87	5.3 (0.4)	5.5 (0.4)	**0.04**	**<0.01**	**0.03**	0.14
Total cholesterol (mg/dL)	150–200	186.3 (33.1)	185.6 (34.4)	0.94	184.7 (33.6)	168.1 (38.4)	0.13	0.30	0.45	0.23
HDL (mg/dL)	45–90	53.8 (13.8)	50.3 (13.8)	0.33	60.8 (14.5)	54.0 (14.7)	0.08	**<0.01**	**0.01**	0.55
LDL (U/L)	70–160	108.6 (30.4)	109.0 (27.1)	0.96	105.0 (26.4)	91.1 (32.4)	0.12	0.11	0.61	0.19
Triglycerides (mg/dL)	30–200	119.7 (45.9)	127.3 (50.3)	0.56	100.1 (74.0)	115.6 (40.5)	**0.04**	0.06	0.11	0.72
Bilirubin (mg/dL)	0.3–1.1	0.7 (0.3)	1.0 (0.9)	**0.02**	0.6 (0.4)	0.6 (0.2)	0.86	**0.03**	0.04	**0.05**
Vitamin D (nmoles/litro)	37.0–160.0	51.2 (33.4)	63.3 (28.1)	**0.02**	60.4 (31.8)	65.0 (25.1)	0.53	0.72	0.36	0.62
Transferrin (mg/dL)	200.0–360.0	245.5 (55.0)	252.0 (34.6)	0.48	232.8 (31.7)	251.4 (22.1)	**0.03**	0.11	**0.04**	0.40
Folic Acid (ng/mL)	3.1–20.5	8.4 (3.6)	9.2 (7.8)	0.60	10.2 (5.9)	7.8 (4.0)	0.20	0.60	0.51	0.19

Data presented as mean (x̄), standard deviation (SD), and *p* values. The significance threshold was set at *p* < 0.05 *. *p* value refers to the comparison of variables’ mean between patients with low-grade MI and SLE using *t*-test or Mann–Whitney test, according to the distribution of the data assessed by Shapiro–Wilk test. Low BMI refers to BMI ≤ 30 kg/m^2^ and high BMI refers to BMI ≥ 30 kg/m^2^. “Group of disease” column is the comparison of variables’ mean between the two inflammatory diseases (without considering BMI status). “BMI levels” column is the comparison of variables’ mean between the low and high BMI. Interaction column means the *p* value of comparison between the two disease groups and the BMI levels (ANOVA 2×2). HDL, High Density Lipoprotein; LDL, Low Density Lipoprotein. *p* value lower than 0.05 are in bold type.

The comparison of BMI levels without considering the type of disease (BMI level column, Table 2) revealed significant differences in HDL-cholesterol, glycated hemoglobin, insulin, and transferrin, being significantly higher in the participants with more BMI, excepting in HDL-cholesterol which was lower. The 2×2 ANOVA interaction analysis (Interaction column, Table 2) showed a trend of significance in bilirubin levels, suggesting that this variable could present a modification of the effect depending on the type of inflammatory disease and BMI status.

Table 3 shows the comparison of inflammatory and coagulation profiles between patients with low-grade MI and SLE, considering the impact of BMI levels. In patients with low-grade MI and high BMI, significant differences in CRP levels were observed. In contrast, patients with SLE and high BMI showed more significant increases in both fibrinogen and CRP. Additionally, regression analysis indicated a significant association between elevated BMI and increased CRP levels (*p* = 0.0003).

**Table 3:** Comparison of inflammatory and coagulation markers among the two types of inflammatory conditions (low-grade MI and SLE) and according to BMI levels.

Variables	Hospital reference values	Low-grade metabolic inflammation (MI)			Systemic lupus erythematosus (SLE)			*p* value		
		Low BMI (*n* = 24)	High BMI (*n* = 46)	*p* value	Low BMI (*n* = 39)	High BMI (*n* = 18)	*p* value	Group of disease	BMI levels	Interaction
RDW (%)	8–14.8	13.7 (0.7)	13.6 (0.8)	0.57	13.7 (1.1)	15.4 (5.1)	0.13	0.13	0.25	**0.03**
Fibrinogen (mg/dL)	200–400	359.5 (95.1)	366.8 (76.4)	0.38	325.8 (79.2)	427.7 (135.3)	**0.01**	0.73	0.01	**0.01**
D-dimer (ng/mL)	0.0–500.0	318.2 (137.1)	329.2 (140.3)	0.71	307.0 (156.6)	422.5 (232.9)	0.16	0.57	0.16	0.12
C-reactive protein (mg/L)	0.1–10	2.5 (2.7)	4.6 (4.3)	**0.02**	3.6 (9.7)	8.6 (10.1)	**0.003**	0.31	**0.05**	0.31
IL-6 (pg/mL)	0–4.4	3.7 (2.6)	3.5 (1.6)	0.30	3.5 (2.0)	3.7 (1.5)	0.15	0.95	0.90	0.65
Platelets (10E3/μL)	150–400	233.71 (43.7)	240.0 (51.1)	0.75	234.1 (64.3)	237.9 (77.6)	0.72	0.81	0.60	0.92
Ferritin (ng/mL)	5.0–204.0	151.4 (96.7)	120.0 (93.3)	0.18	87.0 (77.0)	75.8 (49.3)	0.83	**<0.01**	0.75	0.54

Data presented as mean (x̄), standard deviation (SD), and *p* values. The significant threshold was set at *p* < 0.05*. *p* value refers to the comparison of variables’ mean between patients with low-grade MI and patients with SLE using *t*-test or Mann–Whitney test. According to the distribution of the data assessed by the Shapiro–Wilk test. Low BMI refers to BMI ≤ 30 kg/m^2^ and high BMI refers to BMI ≥ 30 kg/m^2^. “Group of disease” column is the comparison of variables’ mean between the two inflammatory diseases (without considering BMI status). “BMI levels” column is the comparison of variables’ mean between the low and high BMI. Interaction column means the *p* value of comparison between the two disease groups and the BMI levels (ANOVA 2×2). IL-6, Interleukin-6; RDW, Red Cell Distribution Width. *p* value lower than 0.05 are in bold type.

The analysis performed according to type of diseases without considering the BMI revealed significant differences in ferritin levels, showing increased values in low-grade MI group. Additionally, a subgroup analysis based on BMI levels (BMI levels column, Table 3) revealed a trend of significance (*p* = 0.05) for CRP, which was higher in high BMI groups. Moreover, the analysis of the interaction between disease type and BMI levels revealed that RDW and fibrinogen were significantly different in this METAINFLAMMATION cohort, suggesting a modification of the effects for these variables according to the type of inflammatory disease and BMI.

### 3.2 Analysis of alpha and beta diversity of gut microbiota

**Figure 1:**
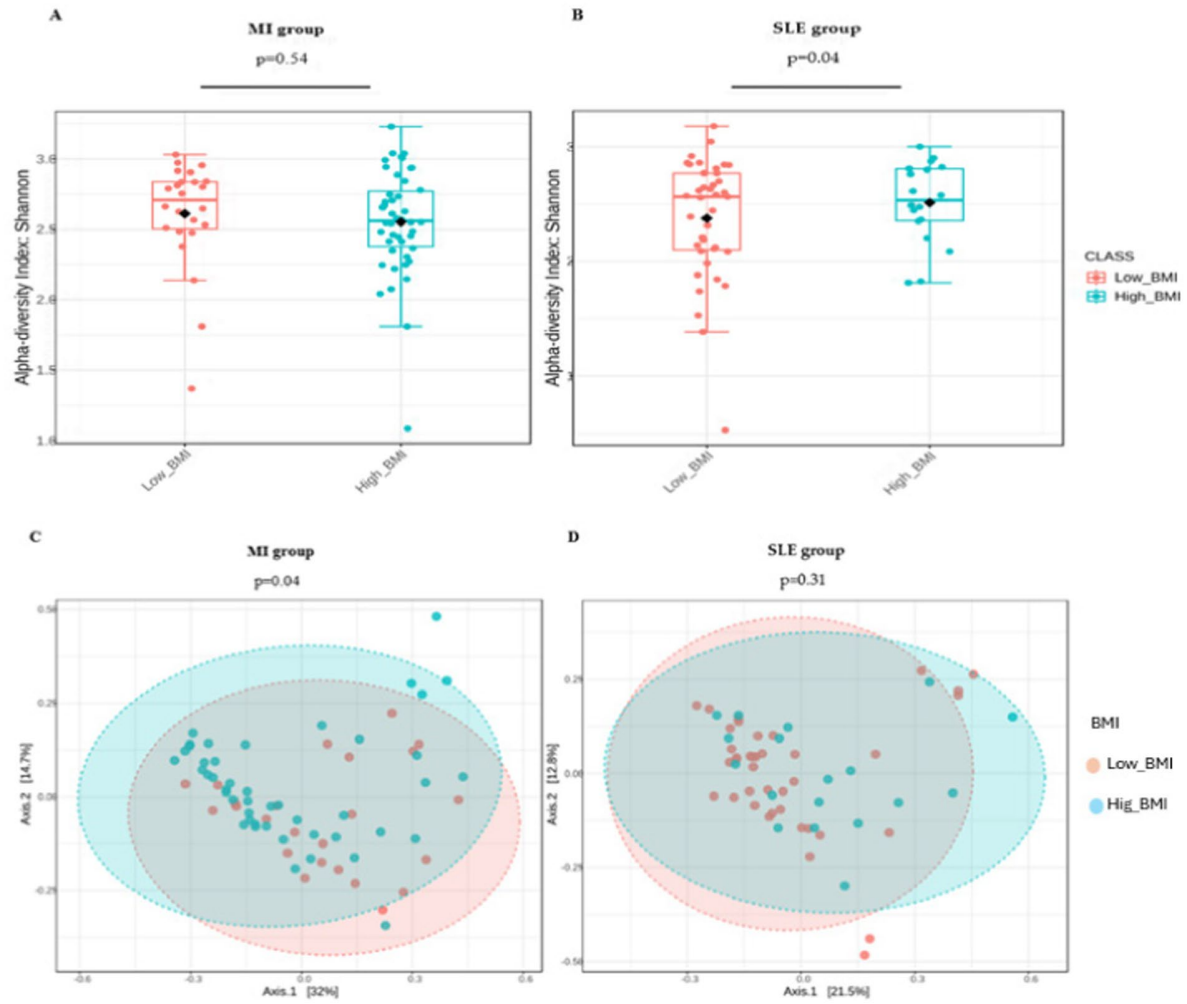
The alpha diversity index (A,B) and principal coordinate analysis (C,D) show the comparison of microbial diversity in patients with low-grade MI and SLE according to BMI levels. Patients with low BMI are represented in orange, and those with high BMI are in blue. In panels (A,B) the differences in box heights and outliers allow comparing the internal diversity of each group (alpha diversity) according to BMI. These findings were verified using a *t*-test. In panels (C,D), the dispersion and spacing between the circles reflect the diversity between groups (beta diversity), where a larger distance implies more pronounced differences in microbial composition between patients with different BMI.

Figure 1 shows the comparison of alpha diversity assessed using Shannon index between patients with low and high BMI in low-grade MI group (Figure 1A) and low and high BMI in patients with SLE (Figure 1B). SLE patients demonstrated significant differences in alpha diversity (*p* = 0.04), while MI patients showed no significant differences in alpha diversity between BMI levels (*p* = 0.54). Analysis indicated that the mean alpha diversity values in the high BMI group was higher than in the low BMI group, as represented by the black dot. The principal coordinate analysis of beta diversity between disease groups using the Bray-Curtis index compared by PERMANOVA was significantly (*p* = 0.04) comparing participants in the low-grade MI group (Figure 1C) with BMI levels, in contrast to the SLE group (Figure 1D) which showed no significant difference.

### 3.3 Analysis of gut microbiota biomarkers and differential abundance between groups

**Figure 2:**
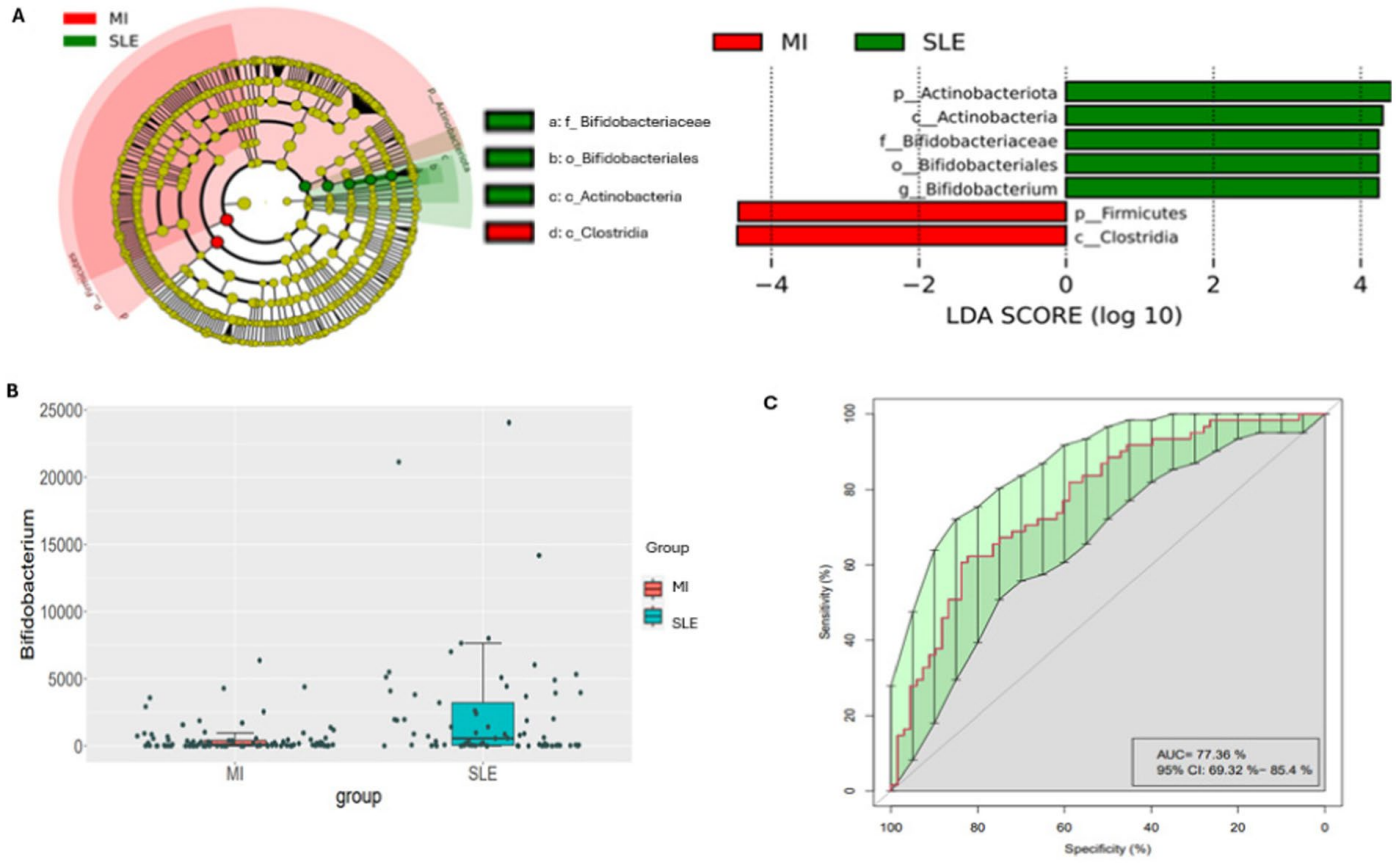
Cladogram and linear discriminant analysis between low-grade MI and SLE participants. Only taxa meeting a *p* < 0.05 and LDA score significant threshold | > 2| are shown. Red bacterial taxa statistically overrepresented in low-grade MI participants; green bacterial taxa overrepresented in participants with SLE (A). A bar plot shows the differential abundance of the *Bifidobacterium* genus, with red boxes for patients with low-grade MI and blue boxes for SLE (B). The ROC analysis demonstrates the model’s ability to differentiate between low-grade MI and SLE with an Area Under the Curve (AUC) of 77.36% and a 95% Confidence Interval of 69.32–85.4%, highlighting the importance of each feature in classification (C). In panel (A), the cladogram and linear discriminant analysis show the bacterial taxa with significant differences. This allows a quick comparison of which bacteria are most common in each disease. Panel (B) highlights the abundance of the genus *Bifidobacterium*, showing that the red and blue colors in the boxes correspond to patients with low-grade MI and SLE, respectively. Panel (C) presents an ROC curve assessing the classification performance of the model, with an AUC of 77.36%, indicating moderate accuracy in distinguishing between low-grade MI and SLE based on bacterial composition.

Firstly, Figure 2A shows the analysis of the most differential taxa between groups of disease. The cladogram and bar chart with LDA score (Figure 2A) highlighted an overrepresentation the genus *Bifidobacterium*, belonging to the Bifidobacteriaceae family, the Bifidobacterial order, the Actinobacteria class and the Actinobacteriota phyla in the participants with SLE, in this cohort. By the contrary, participants with low-grade MI showed an overrepresentation of Firmicutes phyla and Clostridia. In addition, the analysis of differential abundance assessed by EdgeR revealed that participants with SLE presented significantly more abundant *Bifidobacterium* compared to low-grade MI participants in this population (Figure 2B). Likewise, a random forest analysis was performed, and the important plot showed that *Bifidobacterium* was an important bacterial genus for distinguishing between types of diseases. An operational characteristic curve of the receiver (ROC) was performed to validate the results of the Random Forest. The ROC analysis showed an area under the 77.36% curve (AUC) with a 95% confidence interval: 69.32–85.4%. This result indicates a good capacity of the model to distinguish between patients with SLE and low-grade MI, showing the usefulness of identified bacterial biomarkers (Figure 2C), also confirmed in the importance plot showed in supplementary Figure 1.

**Figure 3:**
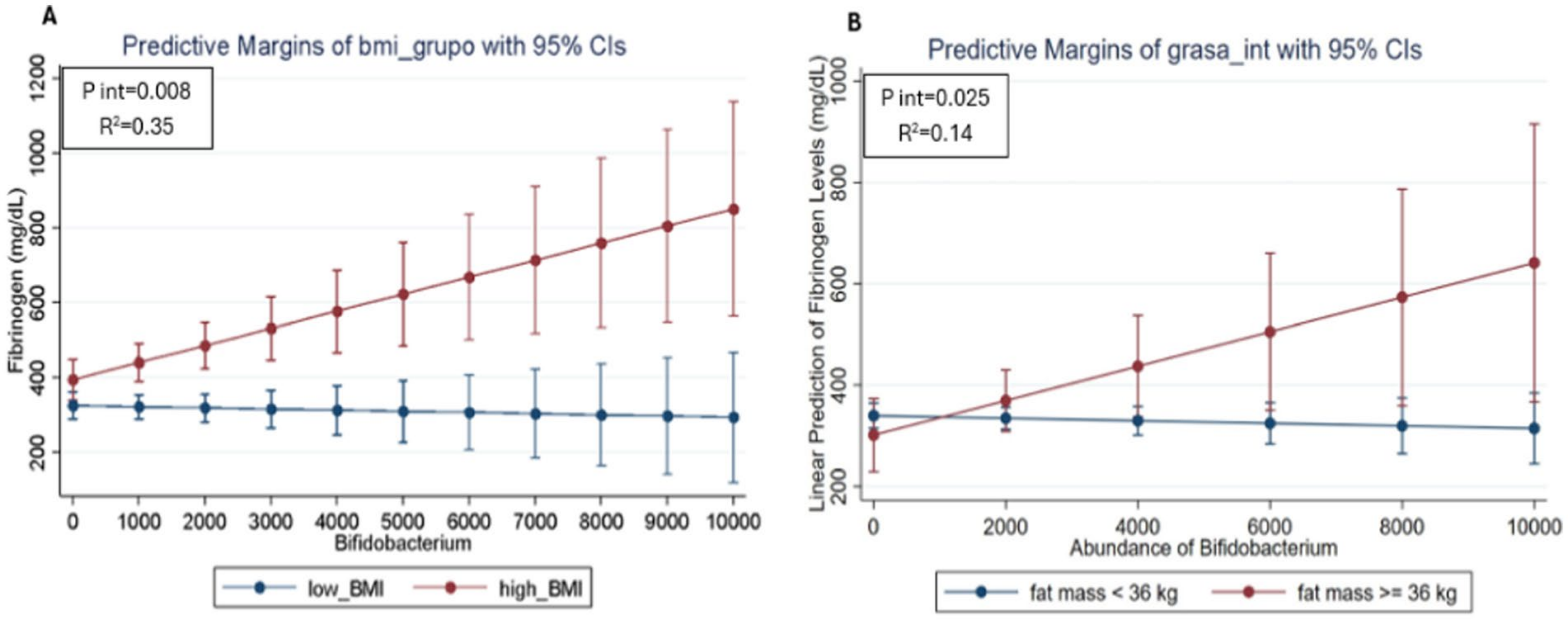
Predicted values of fibrinogen in SLE participants according to the relative abundance of *Bifidobacterium* and BMI status (A) and body fat levels (B) using linear regression models adjusted for age and sex. The red line represents participants with high BMI and the blue line represents participants with low BMI. The divergence between the lines suggests how the relative abundance of *Bifidobacterium* may differentially impact fibrinogen levels depending on BMI and body fat, highlighting the potential role of these factors in inflammation among SLE patients.

Finally, evaluate the relationship between gut microbiota, inflammatory markers and type of disease according to BMI status, a regression model was performed, and a significant interaction were found between patients with the BMI, the genus *Bifidobacterium* and fibrinogen values (*p* = 0.008). The interaction plot (Figure 3A) showed that patients with a high BMI and a greater abundance of *Bifidobacterium* presented high fibrinogen values. On the contrary, participants with high abundance of *Bifidobacterium* but low BMI, showed lower values for fibrinogen, suggesting a modification of the effect on fibrinogen values depending not only on the abundance of *Bifidobacterium*, but also depending on BMI. On the other hand, the second interaction plot (Figure 3B) showed that patients with high body fat and higher *Bifidobacterium* abundance had higher fibrinogen values. In contrast, participants with high *Bifidobacterium* abundance but low fat showed lower fibrinogen values, suggesting a modification of the effect on fibrinogen values depending not only on *Bifidobacterium* abundance, but also on body fat levels (*p* = 0.025). This observation indicates that the high abundance of *Bifidobacterium* seems to modulate both the effects of BMI and body fat on fibrinogen levels in patients with SLE in this cohort.

## 4 Discussion

This study compares patients with two different inflammatory diseases – low-grade MI and SLE – analyzing the differential impact of BMI and body fat, as well as the possible implications of gut microbiota composition in each condition. The findings suggest that gut microbiota may play a key role in modulating inflammation and coagulation, particularly in patients with SLE and high BMI, underscoring the relevance of considering both metabolic and microbial factors in the management of these diseases.

The research shows a relevant intersection between SLE and low-grade MI in shared immuno-inflammatory mechanisms (Pesqueda-Cendejas et al., 2023). When comparing anthropometric and body composition profiles of patients with low-grade metabolic inflammation and SLE, the former had unhealthy indicators of body weight, BMI, skeletal muscle mass and visceral fat, while SLE patients had higher levels of body fat. These results are consistent with previous studies linking obesity to both metabolic inflammation and SLE, noting that elevated BMI in SLE patients is associated with increased severity and risk of complications, highlighting the importance of considering BMI in assessment and treatment (Forsythe et al., 2008; Garcia Cañas et al., 2021; Kono et al., 2021).

Obesity may influence the inflammatory response in patients with autoimmune diseases such as SLE, affecting both disease progression and response to treatment (Karczewski et al., 2021). This underscores the importance of carefully assessing body composition and metabolic status in patients with SLE and other inflammatory diseases, as BMI impacts various anthropometric parameters regardless of disease type. When comparing clinical markers between patients with low-grade MI and SLE, notable differences were observed: patients with low-grade MI and high BMI had elevated CRP levels (Frühbeck, 2015), while in patients with SLE and high BMI, fibrinogen and CRP levels were even higher (Mok et al., 2012). This suggests that, in the context of SLE, high BMI intensifies the inflammatory response and coagulation markers to a greater extent than in low-grade inflammation (Urowitz et al., 2012). The regression analysis reveals that the increase in CRP levels in these patients is mainly due to high BMI and not only due to SLE disease activity *per se* (Kono et al., 2021). Current evidence highlights the relationship between excessive body weight and inflammatory markers, indicating that an elevated BMI may play a significant role in increasing inflammation, resulting in higher CRP levels in patients with SLE (Karczewski et al., 2021; Khanna et al., 2022). Fibrinogen and CRP are relevant biomarkers in SLE because of their association with inflammation and risk of thrombotic events, which are critical aspects of this disease (Bazzan et al., 2015). Although the pathogenesis of vascular disease in SLE is not fully elucidated, evidence suggests that dysregulation of the coagulation system may contribute to both onset and progression disease (Liang et al., 2016). Elevated fibrinogen levels indicate an increased predisposition to thrombosis, as inflammatory responses may induce hypercoagulability through activation of coagulation factors and alteration of anticoagulant mechanisms (Schuett et al., 2015). Furthermore, a correlation between hypercoagulability and clinical manifestations of SLE has been observed, highlighting the potentially adverse effects of disease activity in patients, especially in those with an exacerbated inflammatory response (Liang et al., 2016). Whereas CRP acts as a marker of systemic inflammation and disease activity. In addition, increased RDW, linked to chronic inflammation and cardiovascular risk, highlights the importance of monitoring these markers in the clinical management of SLE (Salvagno et al., 2015). These findings suggest that reducing BMI may help to decrease inflammation and reduce the risk of thrombotic complications, emphasizing the importance of treatment strategies that address both immune and metabolic status in SLE patients.

The diversity of the gut microbiota in patients with SLE and high BMI appears to play an important role in modulating the inflammatory response (Marchesi et al., 2016; Qin et al., 2010). Recent studies have found a higher abundance of the genus *Bifidobacterium* in SLE patients, suggesting that this microorganism may influence the regulation of inflammation in this disease (Clemente et al., 2018; Gavzy et al., 2023; Moya-Pérez et al., 2015; Zhernakova et al., 2016). In contrast, patients with low-grade metabolic inflammation have a microbiota dominated by the phylum Firmicutes and the class Clostridia, rather than *Bifidobacterium* (Scheithauer et al., 2020). The abundance of *Bifidobacterium* in SLE patients has shown an inverse relationship with inflammation, suggesting a potential protective effect against disease progression. Although causality is not yet fully understood, some studies suggest that this increased abundance may be an adaptive response to chronic inflammation in SLE (Wang et al., 2022). These findings highlight the value of microbial profiles as potential differentiating biomarkers in inflammatory diseases and suggest that, with further research, they could serve as useful diagnostic or prognostic tools in the management of SLE and similar inflammatory conditions (Hevia et al., 2014; Mitsuoka, 1990; Rodríguez-Carrio et al., 2017).

The adjusted regression model revealed significant associations between BMI/ body fat, *Bifidobacterium* abundance concerning fibrinogen levels. This finding suggests a complex interaction between gut microbiota, metabolic status and markers of inflammation and coagulation. Previous research has consistently shown that elevated BMI is associated with increased levels of inflammatory markers in patients with autoimmune diseases, as seen in studies indicating the prevalence of obesity in patients with SLE, correlating with increased disease activity and impaired quality of life (Campos-López et al., 2021; Katz et al., 2011; Katz et al., 2011). Our findings support this notion, corroborating that elevated BMI may exacerbate inflammatory processes in SLE. However, the genus *Bifidobacterium* has been documented to have immunomodulatory effects, promoting the production of anti-inflammatory cytokines. For example, one study showed that certain strains of *Bifidobacterium* can decrease proinflammatory cytokines and enhance immune tolerance (Kondo et al., 2010). In contrast, our results indicate that a higher abundance of *Bifidobacterium* is associated with higher levels of fibrinogen, contradicting the idea of a protective role against inflammation. This discrepancy could be explained by the influence of high BMI on the relative abundance of this bacterium. Additionally, patients with active SLE were found to have significantly higher fibrinogen levels compared to those in remission (Sulimai et al., 2022). Our results suggest that this relationship may be mediated by the composition of the gut microbiota and metabolic factors, implying a more complex interaction than previously understood. Previous studies have often isolated factors such as BMI, inflammatory markers and gut microbiota in their analyses, highlighting the role of fibrinogen in coagulation processes without considering the impact of gut microbiota (Cicarini et al., 2020). Current study emphasizes the need to examine these components as interrelated factors, suggesting that traditional approaches may overlook the multifactorial nature of SLE. Regression models indicate that elevated BMI is associated with elevated fibrinogen levels, suggesting a procoagulant and proinflammatory state in SLE patients. These features are clinically relevant, as both obesity and elevated fibrinogen are independent cardiovascular risk factors (Wu et al., 1992). Therefore, it could be speculated implementing strategies to control weight and promote *Bifidobacterium* abundance could be relevant to reduce fibrinogen levels and cardiovascular risk in this population, providing a comprehensive approach to SLE treatment.

This investigation has several significant strengths. Firstly, a comparative analysis between low-grade inflammation and inflammation associated with autoimmune diseases was performed, allowing for a comprehensive assessment of the subject. In addition, a wide range of anthropometric, biochemical, inflammatory and hepatic markers were included, along with an analysis of the composition of the gut microbiota. This provided a comprehensive perspective on the study population and its potential implications for nutrition and personalized medicine. Importantly, all patients received comparable drug treatments according to medical diagnosis, ensuring that the observed variations in inflammatory markers were mainly due to disease activity, rather than differences in treatment prescription. Furthermore, another aspect of this research is the use of BMI as the primary variable in the analyses. While body fat offers a more direct measure of adiposity and related effects, BMI was chosen for the high translational value and ease of interpretation within clinical and research settings. To further understand the relationship between these measures, a correlation analysis between BMI and body fat was performed to assess their association.

However, the study also has limitations. The sample size was relatively small in both groups, which could affect the generalizability of the results, although the results are considered plausible. While we recognize the importance of independently identifying patients with MetS or integrative components, our primary focus was to assess the effects of BMI and body fat on fibrinogen levels with respect to gut microbiota composition. Accordingly, body fat was included as a moderating factor in the regression models between microbiota and coagulation markers. Additionally, our regression model was adjusted for age, sex, BMI, and comorbidities to enhance its accuracy. Given that elevated BMI frequently correlates with MetS components, this assumption supports our use of BMI categorization for this study. Finally, it is important to highlight that the results of this investigation, while revealing significant associations between *Bifidobacterium* abundance, BMI/body fat, and fibrinogen levels, should be interpreted as associative trends rather than indicative of causation. The observed interactions may be influenced by confounding factors, including diet, physical activity, and other health determinants that have not been controlled in this analysis, while type I and II errors cannot be discarded.

This research evidences a modification of the effect on fibrinogen levels involving the *Bifidobacterium* genus and high BMI levels in SLE patients, highlighting the complexity of interactions between coagulation markers, gut microbiota and the host’s immune response, reflecting the complexity of SLE as an autoimmune disease. Furthermore, the findings highlight the importance of considering gut microbiota, BMI and fibrinogen levels as interrelated factors in the assessment and treatment of SLE, suggesting that further research into these mechanisms could lead to accurate clinical management of SLE patients.

## Data Availability

The raw data supporting the conclusions of this article will be made available by the authors, without undue reservation.

## Footnotes

^1^http://ccb.jhu.edu/software/FLASH/

^2^https://github.com

^3^https://www.arb-silva.de/

^4^https://github.com/torognes/vsearch/

^5^https://qiime2.org

^6^https://www.R-project.org/

^7^https://www.microbiomeanalyst.ca/

^8^http://huttenhower.sph.harvard.edu/galaxy/

^9^http://www.stata.com
