# Supplementary material for: Body mass index and fat influences the role of Bifidobacterium genus in lupus patients concerning fibrinogen levels
Source: Front Microbiol. 2024 Nov 25;15:1471177. doi: 10.3389/fmicb.2024.1471177 (PMC11625790; doi:10.3389/fmicb.2024.1471177)
Supplement: Supplementary file 1 [file Supplementary_file_1.docx]

***Supplementary Material***

**Fat content influences the role of *Bifidobacterium* genus in lupus patients concerning fibrinogen levels**

**Lourdes Chero-Sandoval ^1,2^, Andrea Higuera-Gómez ^1^, Begoña de Cuevillas^1,2^, Raquel Castejón^5^, María Martínez-Urbistondo^5^, Susana Mellor- Pita^5^, Víctor Moreno Torres^5^; Daniel de Luis ^3^, Amanda Cuevas-Sierra* ^1^, J. Alfredo Martínez ^1,3,4^**

*** Correspondence:** Corresponding Author: amanda.cuevas@alimentacion.imdea.org

## 1. Supplementary Figures


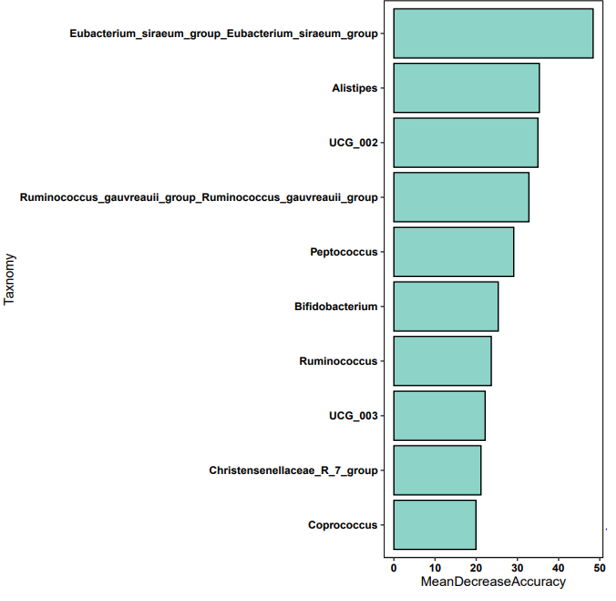


**Supplementary Figure 1.** The important plot from random forest analysis showing the ranking of importance for each variable to discriminate between types of diseases.
